# The analysis of endocrine disruptors in patients with central precocious puberty

**DOI:** 10.1186/s12887-019-1703-4

**Published:** 2019-09-07

**Authors:** Mo Kyung Jung, Han Saem Choi, Junghwan Suh, Ahreum Kwon, Hyun Wook Chae, Woo Jung Lee, Eun-Gyong Yoo, Ho-Seong Kim

**Affiliations:** 1 Department of Pediatrics, CHA Bundang Medical Center, CHA University, Seongnam, South Korea; 2 Department of Pediatrics, Severance Children’s Hospital, Endocrine Research Institute, Yonsei University College of Medicine, Seoul, South Korea

**Keywords:** Central precocious puberty, Phthalates, Bisphenol A

## Abstract

**Background:**

A few studies have reported a positive association between phthalate exposure and pubertal timing, but several conflicting reports exist. The main objective of the study was to determine whether phthalate exposure was associated with central precocious puberty in girls.

**Methods:**

This was a multicenter case-control study wherein 47 girls with central precocious puberty (CPP) and 47 controls (26 pre-pubertal girls and 21 pubertal girls) were enrolled. No obese girls were included. Five phthalate metabolites (creatinine adjusted) and bisphenol A (BPA) were measured in the first spot urine samples of these 94 girls in the early morning.

**Results:**

The median values of monobenzyl phthalate (MBzP), mono-2-ethyl-5-carboxypentyl phthalate (MECPP), mono-2-ethyl-5-hydroxyhexyl phthalate (MEHHP), mono-2-ethyl-5-oxohexyl phthalate (MEOHP), and mono-n-butyl phthalate (MnBP) were 3.1, 29.3, 18.0, 15.4, and 25.2 μg/g creatinine in the CPP group, 4.3, 53.7, 35.7, 29.1, and 66.0 μg/g creatinine in the pre-pubertal control group, and 1.7, 28.7, 21.4, 12.1, and 33.3 μg/g creatinine in the pubertal control group, respectively.

The urinary concentration of the five phthalates was significantly lower in the CPP group than in the pre-pubertal control group (*P* < 0.001). Conversely, there was no significant difference in the urinary concentration of the five phthalates between the CPP and pubertal control groups (*P* values: 0.077 for MBzP, 0.733 for MECPP, 0.762 for MEHHP, 0.405 for MEOHP, and 0.981 for MnBP). In addition, the BPA level was not significantly different between the CPP and pubertal control groups (BPA median values: 0.63 μg/g creatinine, the CPP group; 1.7 μg/g creatinine, the pubertal control group; *P* value = 0.092)*.*

**Conclusions:**

Our study showed that there was no significant difference in the urinary phthalate levels between the CPP and pubertal control groups. Moreover, phthalate metabolites were significantly lower in the CPP group than in the pre-pubertal control group. Further investigation about endocrine disruptors and pubertal progression is needed.

## Background

Precocious puberty is defined as the development of secondary sexual characteristics earlier than two standard deviations of the mean value [1]. In recent times, children have been attaining sexual maturity earlier than they would in the past, and the incidence of precocious puberty is rising worldwide [2]. Central precocious puberty (CPP) is the result of precocious activation of the hypothalamic–pituitary–gonadal axis, and the majority of CPP is idiopathic.

There has been considerable concern regarding the presence of endocrine-disrupting chemicals (EDCs) in the environment, which supposedly disturb the onset and progression of pubertal development [3]. Phthalates are synthetic chemicals that can provide flexibility and durability to polyvinyl chloride products and are present in a wide variety of consumer products, including food packaging, plastic devices, toys, and cosmetics. The association between exposure to phthalates and pubertal onset has been explored, and the results were inconsistent [4, 5]. Moreover, in experimental research, phthalates exhibit both agonist and antagonist effects, suggesting that pubertal development may be accelerated or delayed depending the on timing, dose, and various other factors in female rats [6].

Therefore, we assessed the urinary concentrations of phthalate and bisphenol A (BPA) in girls with CPP and control subjects to investigate the association between exposure to phthalate and development of puberty in Korean girls.

## Methods

### Study design and population

This case-control study was conducted at the Division of Pediatric Endocrinology, Severance Hospital and Bundang CHA Medical Center from 2015 to 2018. We enrolled 47 CPP patients and 47 healthy controls (26 pre-pubertal and 21 pubertal controls). All individuals were analyzed for urinary phthalates and BPA. All participants were asked to fill out questionnaire requesting the following data: personal information including where they live in a city or rural area, usage habits, and dietary habits. The girls with CPP and the controls lived in the same urban area, and there were no specific eating habits or exposures to other polluting materials including the use of plastic packaging. Patients were identified as having idiopathic CPP if they satisfied the following classical diagnostic criteria: (1) the onset of breast development (Tanner stage B2 or above) before 8 years of age, (2) a peak luteinizing hormone (LH) level of 7 IU/L in the standard intravenous gonadotropin-releasing hormone (GnRH) stimulation test, and (3) no evidence of hypothalamic–pituitary organic lesions, confirmed by magnetic resonance imaging. Subjects were excluded if they had any additional condition that could affect the onset of puberty, such as hypothyroidism or congenital adrenal hyperplasia. Healthy controls were recruited when children visited the clinic for growth assessment. The inclusion criteria for pre-pubertal controls were (1) 5 to 8 years of age (Tanner stage 1), and (2) bone age (BA) not advanced 1 year more than the chronological age (CA), and (3) no evidence of systemic illness or endocrinopathy. In addition, pubertal healthy controls (1) were 10 to 12 years old (Tanner stage 2 or above), (2) had BA not advanced 1 year more than CA, and (3) showed no evidence of systemic illness or endocrinopathy. Obese children were also excluded. This study was approved by the Institutional Review Board (IRB) of Severance Hospital (No.2015–0917-007) and Bundang CHA Medical Center (No.2017–09-029).

### Clinical information and specimen collection

Anthropometric measurements were performed by well-trained physicians, and height was measured using a Harpenden stadiometer. BA was assessed using the Greulich–Pyle method by the same observer [7]. Growth parameters, such as height and body mass index (BMI), were expressed as a standard deviation score (SDS), which was calculated using the Korean children and adolescents growth standard [8].

The first spot urine samples were collected in the early morning of the appointment day for all participants. Then, 10 mL of urine obtained from each subject was stored in a polypropylene urine collection cup at − 20 °C until assayed (polypropylene is not reported to contain detectable levels of phthalate).

### Analysis of urinary phthalates and BPA

Five phthalate metabolites (adjusted for creatinine), namely monobenzyl phthalate (MBzP), mono-2-ethyl-5-carboxypentyl phthalate (MECPP), mono-2-ethyl-5-hydroxyhexyl phthalate (MEHHP), mono-2-ethyl-5-oxohexyl phthalate (MEOHP), and mono-n-butyl phthalate (MnBP), and BPA were measured. Briefly, after an aliquot (1.0 mL) of urine sample was enzymatically hydrolyzed and purified by solid-phase extraction, the phthalate metabolites in the urine were resolved by reversed-phase ultra-performance liquid chromatography, detected by electrospray ionization tandem mass spectrometry, and quantified by an isotope internal standard curve method [9].

In detail, HPLC-grade ethyl acetate was purchased from Burdick & Jackson (Muskegon, MI). Ammonium acetate (97.0% powder) was purchased from Junsei Chmical co., Ltd. (Tokyo, Japan). β-Glucuronidase (≥ 85,000 units/mL) from *Helix pomatia* (Type H-2) and Bovine Serum Albumin (≥ 96.0% powder) were obtained from Sigma–Aldrich (St. Louis, MO, USA). Urine sample was fortified with 50 μL of internal standard (200 ng/mL, 9 types mixer each phthalate metabolites-^13^C_12_) spiking solution, 1 mL of 2 M ammonium acetate buffer solution (1.54 g of ammonium acetate / 10 mL HPLC-grade water) and 20 μL of- β-Glucuronidase from *Helix pomatia* source. These samples were incubated with overnight at 37 °C. And then, these samples were extracted twice with 4 mL of ethyl acetate. These samples were gently shaken a few times and separate organic layer from non-polar fat layer by a centrifugal at 4000 rpm for 15 min. The chromatographic separation was performed on a CAPCELL PAK C_18_ MG II column (3.0 mm × 150 mm, 3 μm) from Shiseido co. Ltd. (Tokyo, Japan). Target compound was performed with an Agilent 6430 Triple Quad liquid chromatograph mass spectrometer equipped with Agilent 1200 series HPLC system (Agilent Technologies Inc., Santa Clara, CA). These results were analyzed by Eurofins Korea Analytic Service Co. Ltd.

### Statistical analysis

Statistical analysis of the results was performed using IBM SPSS Statistics ver. 25.0 (IBM Co., Armonk, NY, USA). All data were expressed as mean ± standard deviation, and the paired t-test and ANOVA test were applied to compare the data. A *P*-value of < 0.05 was considered significant.

## Results

### Clinical characteristics

Clinical characteristics of all participants are shown in Table 1. In the CPP group, the CA and BA were 8.53 ± 0.65 years and 10.43 ± 0.68 years, respectively. The CA and BA were 7.03 ± 1.37 and 6.51 ± 1.93 in the pre-pubertal control group and 11.18 ± 1.00 years and 11.39 ± 1.12 years in the pubertal control group, respectively. The height SDS was significantly different among these groups (*P* < 0.001). BMI was not different between the groups. In the analysis of sex hormone, the basal LH levels were 0.65 ± 0.56 mIU/mL in the CPP group and 1.32 ± 1.43 mIU/mL in the pubertal control group. The mean of LH peak of GnRH stimulation in the CPP group was 14.3 ± 9.4 mIU/mL. In addition, the kisspeptin levels were analyzed (data not shown). The levels were 491.0 ± 198.7 pg/mL in the CPP group and were 496.1 ± 314.4 and 2151.4 ± 1137.8 pg/mL in the pre-pubertal and pubertal control groups, respectively. The value for the pubertal control group was statistically different from the values for the other two groups (*P* < 0.001).

**Table 1:** Clinical and biochemical characteristics of CPP and controls

Variable	CPP group (*N* = 47)	Pre-pubertal Control group (*N* = 26)	Pubertal Control group (*N* = 21)	*P* value
CA (years)	8.53 ± 0.65	7.03 ± 1.37	11.18 ± 1.00	< 0.001
BA (years)	10.43 ± 0.68	6.51 ± 1.93	11.39 ± 1.12	< 0.001
Height (cm)	131.87 ± 5.84	113.41 ± 8.47	141.05 ± 9.34	0.002
Height SDS	0.23 ± 0.97	− 1.04 ± 0.66	−0.73 ± 0.86	< 0.001
BMI (kg/m^2^)	17.35 ± 2.67	15.02 ± 1.72	17.36 ± 2.49	0.06
Tanner stage	2.5 ± 0.50 (between stage 2–3)	1.0 ± 0.0 (stage 1)	3.0 ± 0.78 (stage 3)	< 0.001
MPH (cm)	159.6 ± 3.99	158.2 ± 3.17	160.8 ± 4.23	0.066
Basal LH (mIU/mL)	0.65 ± 0.56	0.02 ± 0.35	1.32 ± 1.43	< 0.001
Basal FSH (mIU/mL)	3.71 ± 1.52	1.72 ± 0.62	4.92 ± 2.54	< 0.001
Basal E2 (pg/mL)	below than 8	below than 8	17.3 ± 3.66	
LH peak (mIU/mL)	14.3 ± 9.4			
FSH peak (mIU/mL)	20.4 ± 8.8			

Data are presented as mean ± standard deviation. *CPP* central precocious puberty, *CA* chronologic age, *BA* bone age, *BMI* body mass index, *MPH* mid-parental height, *LH* luteinizing hormone, *FSH* follicle-stimulating hormone, *E2* estradiol

### Urinary concentration of phthalates and BPA

The urinary concentrations of the five phthalates and BPA were obtained and creatinine-adjusted (Table 2). The median values of MBzP, MECPP, MEHHP, MEOHP, and MnBP were 3.1, 29.3, 18.0, 15.4, and 25.2 μg/g creatinine in the CPP group, 4.3, 53.7, 35.7, 29.1, and 66.0 μg/g creatinine in the pre-pubertal control group, and 1.7, 28.7, 21.4, 12.1, and 33.3 μg/g creatinine in the pubertal control group, respectively.

**Table 2:** Urinary concentrations of phthalates and BPA in girls with CPP and control groups

	CPP (*N* = 47)	Pre-pubertal control (*N* = 26)	*P value*	CPP (*N* = 47)	Pubertal control (*N* = 21)	*P value*
MBzP (μg/g creatinine)	3.1 (1.8–4.9)	4.3 (3.3–11.1)	*0.026*	3.1 (1.8–4.9)	1.7 (0.64–4.6)	*0.077*
MECPP (μg/g creatinine)	29.3 (22.1–44.7)	53.7 (35.1–86.4)	*< 0.001*	29.3 (22.1–44.7)	28.7 (22.7–45.6)	*0.733*
MEHHP (μg/g creatinine)	18.0 (14.6–28.5)	35.7 (23.0–54.3)	*0.001*	18.0 (14.6–28.5)	21.4 (13.5–29.4)	*0.762*
MEOHP (μg/g creatinine)	15.4 (12.1–25.0)	29.1 (18.7–35.9)	*< 0.001*	15.4 (12.1–25.0)	12.1 (8.0–17.8)	*0.405*
MnBP (μg/g creatinine)	25.2 (10.9–151.0)	66.0 (39.4–106.1)	*< 0.001*	25.15 (10.92–150.98)	33.3 (25.2–46.2)	*0.981*
Bisphenol A (μg/g creatinine)	0.63 (0.4–1.1)	2.0 (0.93–3.5)	*< 0.001*	0.63 (0.4–1.1)	1.7 (1.1–3.3)	*0.092*

Data are presented as median and interquartile range. *MBzP* monobenzyl phthalate, *MECPP* mono-2-ethyl-5-carboxypentyl phthalate, *MEHHP* mono-2-ethyl-5-hydroxyhexyl phthalate, *MEOHP* mono-2-ethyl-5-oxohexyl phthalate, *MnBP* mono-n-butyl phthalate

The urinary concentrations of all phthalate metabolites were lower in the CPP group than in the pre-pubertal control group (*P* = 0.026 for MBzP; *P* = 0.001 for MEHHP; *P* < 0.001 for MECPP, MEOHP, and MnBP). Furthermore, all phthalate metabolites had higher levels in the pre-pubertal group than in the CPP group. Conversely, on comparing the CPP group and pubertal control group, the urinary concentrations of the five phthalate metabolites did not show significant difference (*P* = 0.077 for MBzP, 0.733 for MECPP, 0.762 for MEHHP, 0.405 for MEOHP, and 0.981 for MnBP). In addition, urinary concentration of BPA showed same pattern between groups, implying that the BPA level was not significantly different between the CPP and pubertal control groups. The median value of BPA was 0.63 μg/g creatinine in the CPP group and 1.7 μg/g creatinine in the pubertal control group (*P* = 0.092*).*

## Discussion

The incidence of CPP is rising worldwide, particularly in Korean children [2, 10, 11]. It is undetermined why the incidence of CPP is increasing in Korean children. Factors contributing to the timing of puberty include genetic and environmental factors [12]. Evidence for genetic regulation of pubertal timing is supported by the observations that high correlation of the onset of puberty seen within families, within racial/ethinic groups, and between monozygotic compared to dizygotic twins [13]. Secular trends in the timing of puberty over the past decades indicate that environmental factors also influence the timing of puberty. It is possible that environmental factors, such as obesity, nutrition, dietary habits, physical activity, and exposure to EDCs play an important role in pubertal timing through directly or interacting the genes regulating the puberty. Abrupt increase in the incidence of CPP in Korean children suggests that environmental factors, such as EDCs, are involved in the development of CPP in Korea. Consequently, there have been considerable concerns with the influence of EDCs, such as phthalates and BPA, on precocious puberty because the use of these chemicals is widespread, making the exposure of people to these chemicals very easy and likely. However, there have been no consistent reports suggesting that phthalates and BPA promote the early onset of puberty. Recent studies exploring the association between phthalate [14–18] or BPA [19–21] exposure and pubertal timing are described in Table 3.

**Table 3:** Summary of recent studies on association between phthalates or BPA and puberty

Author (year), country	Subjects	Phthalates or BPA	Results
Colon et al. [14] (2000) Puerto Rico	41 thelarche patients, 35 controls	DBP, BBP, DEP MEHP	Elevated serum phthalates in premature thelarche
Chou et al. [15] (2009) Taiwan	26 CPP, 30 premature thelarche 33 controls	MMP, MBuP MBzP, MEHP	Urinary levels of MMP were higher in premature thelarche (but not in CPP group) None of the phthalates showed association with true gonadotropin-dependent puberty
Lomenick et al. [16] (2010) USA	28 CPP girls, 28 age-matched controls	MBP, MBzP MCPP, MECPP MEHHP, MEHP MEOHP, MEP MiBP	Phthalate exposure is not associated with precocious puberty in female children.
Chen et al. [17] (2013) Taiwan	73 CPP girls, 31 controls	MMP, MEP MBP, MBzP MEHP, MEHHP MEOHP	All seven urinary phthalate metabolite levels in the CPP group were significantly higher (*P* < 0.05) than in prepubescent controls.
Srilanchakon et al. [18] (2007) Thailand	42 precocious puberty, 17 early puberty, 77 age-matched controls	MMP MEP	Urinary MEP concentration was higher in girls with precocious puberty than in controls
Durmaz et al. [19] (2014) Turkey	28 CPP non-obese girls, 25 controls	BPA	Urinary BPA levels in CPP group were higher compared to the control
Özgen et al. [20] (2016) Turkey	28 CPP, 28 premature thelarche, 22 prepubertal controls	BPA	Urinary BPA levels did not differentiate between groups
Chen et al. [21] (2018) China	136 CPP, 136 age-, BMI-matched controls	BPA	BPA exposure was associated with increased incidence of CPP

*CPP* central precocious puberty, *DBP* dibutyl phthalate, *BBP* benzylbutyl phthalate, *DEP* diethyl phthalate, *MEHP* mono- (2-ethylhexyl) phthalate, *MMP* monomethyl phthalate, *MBuP* monobutyl phthalate, *MBzP* monobenzyl phthalate, *MBP* mono-n-butyl phthalate, *MCPP* mono-3-carboxypropyl phthalate, *MECPP* mono (2-ethyl-5-carboxypentyl) phthalate, MEHHP, mono (2-ethyl-5-hydroxyhexyl) phthalate, *MEOHP* mono (2-ethyl-5-oxohexyl) phthalate, *MEP* monoethyl phthalate, *MiBP* mono-isobutyl phthalate, *BPA* bisphenol A

Some studies have reported associations between phthalate exposure and early onset of puberty. Colon et al. reported higher serum diethylhexyl phthalate levels in 41 thelarche patients than in 35 age-matched controls [14]. They suggested that phthalates with weak estrogen activity may disrupt the biologic system if they act at critical periods of development. In addition, in a recent study in Taiwan, all seven urinary phthalates were significantly higher in the CPP group than in pre-pubertal controls [17]. Chen et al. also analyzed these groups using estrogen receptor binding effect indices, and the results were similar [17]. Meanwhile, a recent study in the US showed no difference in nine urinary phthalates between girls with CPP and pre-pubertal controls, suggesting that phthalate exposure is not associated with CPP [16]. They proposed some possibilities that phthalates have no significant estrogen effect, or the corresponding phthalate metabolites, which are formed rapidly in vivo from their parent compound, have no significant clinical estrogenic effect, although some phthalates have weak estrogenic activity in vitro [22]. In another study in Taiwan, monomethyl phthalate (MMP) concentrations were higher in the premature thelarche group (non-gonadotropin-dependent group, normal variant) than in the control group but were not significantly different from the concentrations in the CPP group [15]. The level of any phthalate (including MMP) was not significantly different between the CPP and control groups, and thus, the levels do not suggest an association with true gonadotropin-dependent puberty. Additionally, in a study conducted in Shanghai, Xie et al. found that phthalate exposure delayed the puberty in men and that urinary phthalate concentrations were significantly associated with constitutional delay of growth and puberty [23]. In addition, the anti-androgenic effect of phthalates on testosterone production has been proven in animal experiments and in vitro [24, 25]. Association between BPA exposure and development of CPP is also controversial. Some studies reported that urinary levels of BPA in CPP group were significantly higher compared to the controls [19, 21], while other study did not show any difference between groups [20].

In our study, urinary concentrations of phthalate metabolites and BPA were lower in girls with CPP than in pre-pubertal controls and were similar to those of pubertal controls. Our finding of comparable levels of phthalates and BPA in CPP and pubertal control groups suggests that phthalates and BPA are not associated with the development of CPP. The lower urinary concentrations of phthalates and BPA in girls with CPP than in pre-pubertal controls in our study are not consistent with previous studies. Our assumption is that lower urinary concentrations of phthalates and BPA in girls with CPP reflect the increased excretion of these metabolites rather than lesser exposure to them during the progress of puberty. Urinary concentration of phthalates and BPA can be influenced by the extent of exposure to them and their excretion rate from the body. According to the National Health and Nutrition Examination Survey (NHANES), the urine levels of all phthalates were the highest in 6–11-year age group among all the groups (6–11 years, 12–19 years, 20–59 years, and 60–80 years), except for those of mono-ethyl phthalate (MEP) [26]. In Korean Environmental Health Survey in Children and Adolescents (KorEHS-C) comprising 351 students, a significant decreasing trend of phthalate concentration with increasing age was consistent with a US study [27]. In our study, urinary concentrations of phthalate metabolites and BPA were lower in pubertal normal controls than in pre-pubertal controls. These findings suggest that the excretion of urinary phthalates and BPA increases with age and pubertal progression. However, it should be determined whether the excretion of phthalates and BPA from the body increases with age and pubertal progression; further, the involved mechanism should be elucidated through further investigations.

There are several possible reasons for the results of the effect of phthalate exposure on puberty onset being inconsistent among previous studies as well as in our present study. First, the discrepancy in the results may be related to the timing of phthalate exposure. It has been hypothesized that puberty comprises a series of network of processes which are regulated by numerous genes and several environmental factors [28]. Therefore, it is possible that humans are the most vulnerable to being affected by phthalate exposure only at a certain time period (window period) or age range, or phthalate exposure does not cause precocious puberty in any time. Second, we do not know the normal reference range of phthalates and BPA and the mechanisms that affect their metabolism. In addition, many factors, such as age and BMI may affect the level of phthalates and BPA. The urine levels of phthalate metabolites reportedly decrease with increasing age [26, 27]. BMI and waist circumference can also affect the levels of phthalates, which are highly variable in children by age and developmental status and related in part to the timing of adiposity rebound [29]. Third, there is a possibility that technical factors, such as analysis methods and the type of sample container may lead to different results. Lastly, because the half-life of any phthalate is very short, there is a possibility that the urinary concentration of phthalates has changed even with a lifestyle change of just a few days. Recently, the Korean government has begun to regulate the concentration of phthalates in children’s products (including toys and all synthetic resins used by children); the urinary concentration of phthalates could change after this regulation by the government comes into effect.

Our study has some limitations. The number of patients is insufficient to detect a significant difference in phthalate levels between the groups. In addition, our phthalate measurements were from a single urine sample despite the short half-life of phthalates. Further, heterogeneity owing to regional diversity and differences in living habits should be considered. Strengths of our study include that to our knowledge, this is the first study involving the analysis of urinary phthalates between the CPP and control groups in Korean girls.

## Conclusions

We found no significant differences in urinary phthalates and BPA between girls with CPP and pubertal controls. Rather, phthalate metabolites and BPA were relatively higher in the pre-pubertal group than in the CPP group. Prospective, longitudinal, large-scale human studies are needed.

## Data Availability

The datasets used and/or analyzed during the current study are available from the corresponding author on reasonable request.

## Abbreviations

BBP: Benzyl butyl phthalate
BMI: Body mass index
BPA: Bisphenol A
CPP: Central precocious puberty
DBP: Dibutyl phthalate
DEP: Diethyl phthalate
EDCs: Endocrine-disrupting chemicals
GnRH: Gonadotropin-releasing hormone
KorEHS-C: Korean Environmental Health Survey in Children and Adolescents
LH: Luteinizing hormone
MBzP: Monobenzyl phthalate
MCPP: Mono-3-carboxypropyl phthalate
MECPP: Mono-2-ethyl-5-carboxypentyl phthalate
MEHHP: Mono-2-ethyl-5-hydroxyhexyl phthalate
MEHP: mono- (2-ethylhexyl) phthalate
MEOHP: Mono-2-ethyl-5-oxohexyl phthalate
MEP: Mono-ethyl phthalate
MiBP: Mono-isobutyl phthalate
MMP: Monomethyl phthalate
MnBP: Mono-n-butyl phthalate
NHANES: National Health and Nutrition Examination Survey
SDS: Standard deviation score
